# Fellowship Training Trends Among Spine Neurosurgeons in Florida: A Cross-Sectional Analysis

**DOI:** 10.7759/cureus.111762

**Published:** 2026-06-29

**Authors:** Maria Fioletova, Umangpreet Gill, Alexandra Lugo, Evelyn C Echevarria Cruz, Julieanne P Sees

**Affiliations:** 1 Surgery, Nova Southeastern University Dr. Kiran C. Patel College of Osteopathic Medicine, Davie, USA; 2 Internal Medicine, Nova Southeastern University Dr. Kiran C. Patel College of Osteopathic Medicine, Davie, USA; 3 Haub School of Business, St. Joseph's University / American Osteopathic Association, Chicago, USA

**Keywords:** academic neurosurgery, fellowship training, florida neurosurgeons, geographic distribution, neurosurgical workforce, spine fellowship, spine neurosurgery, subspecialization trends, women in neurosurgery, workforce diversity

## Abstract

Introduction: Neurosurgical fellowships to obtain additional surgical training after residency are a career option widely available to the newer generation of neurosurgeons. This study aims to analyze current trends in fellowship training among spine neurosurgeons in Florida and factors that influence the choice of fellowship based on year, location, and academic trajectory.

Methods: This retrospective cross-sectional study collected demographic data and bibliometric information on neurosurgeons specializing in spine, comparing those with fellowship training and those without. The information was gathered from the American Association of Neurological Surgeons and the Florida Department of Health databases.

Results: A total of 113 spine-specialized neurosurgeons with active medical licenses were identified in the state of Florida at the time of this study in 2026. A proportion (23.53%; n = 12) of Florida-based spine neurosurgeons obtained their fellowship training in Florida, most commonly at the University of Miami/Jackson Health System. Out of a total of 113, only two (1.77%) spine neurosurgeons are female, and four (3.54%) have osteopathic doctoral degrees (D.O.). In 1960-1964 and 1975-1979, none (n = 0) had completed a spine fellowship, while 33.33% (n = 1) had done so in 1970-1974. The proportion was 28.57% (n = 2) in 1980-1984 and 16.67% (n = 2) in 1985-1989. An overall increase was observed in subsequent years: 23.53% (n = 4) in 1990-1994, 47.83% (n = 11) in 1995-1999, 50.00% (n = 4) in 2000-2004, 62.50% (n = 5) in 2005-2009, and 75.00% (n = 6) in 2010-2014. The highest percentage was observed in 2015-2019 at 73.33% (n = 11), followed by 66.67% (n = 6) in 2020-present.

Conclusion: Over the past 60+ years, there has been an increase in the number of Florida-based spine neurosurgeons pursuing fellowship training, with the highest percentage seen in those graduating from 2015-2019. However, there is still a low representation of female and osteopathic spine neurosurgeons in Florida. This growing trend of spine-specialization highlights a potential shift in neurosurgery career trajectories and has important implications for future workforce planning and patient care in spine surgery.

## Introduction

Surgical training has evolved in the United States, leading to an expansion of surgical subspecializations. According to JAMA Health Forums, a study found that subspecialists in neurosurgery rose from 66% to 77% in 2021. The younger generation of doctors is more inclined to narrow their scope of practice and subspecialize. It was found that 84% of neurosurgeons from 2005 to 2010 subspecialized, while only 63% in resident cohorts from 1975 to 1979 [1]. As surgeons trend toward pursuing fellowships, the number of surgeons performing a wide range of procedures has declined, which could impact the geographic access to care. Another study found that residents from the top 40 National Institutes of Health (NIH)-funded institutions were more likely to complete fellowships when compared to residents from small cities [2]. This further shows the role of geographic location in impacting the likelihood for neurosurgery residents to pursue a fellowship.

Spine surgery has also seen an increase in complexity, calling for more advanced training. Integration of artificial intelligence, advanced visualization systems, and robotic surgery has contributed to the increase in subspecializations. In addition, the shift toward minimally invasive spine surgery (MISS) and endoscopic procedures while managing both precision demands and anatomical variants presents new challenges for those pursuing spine surgery. These factors have contributed to the need for advanced training to properly integrate technology. Simulation-based training using virtual reality, for example, has become more popular in the field [3,4]. While this shift toward visualization systems has played a role in reducing surgeon fatigue and improving ergonomics, the shift requires further training [5]. This has contributed to the increased need for fellowship-trained spine neurosurgeons. With evidence indicating that fellowship-trained spine neurosurgeons are better equipped to manage the complexities behind technological innovation and effective patient care, the modern expectation is shifting toward pursuing a fellowship. However, there are disparities in those numbers. According to the Journal of Neurosurgery, out of a total of 1,641 academic neurosurgeons, 1,403 were fellowship-trained; 89.9% were men and only 10.1% were women. The majority of women have completed fellowships in pediatrics and neuro-oncology, while significantly more men completed spine fellowships [6]. It is observed that both geographical location and sex are factors in pursuing fellowship training. 

Spine fellowship following neurosurgical training remains the most popular subspecialization; it accounts for 16.04% of all fellowships among the academic neurosurgeons studied by the World of Neurosurgery Journal. The same article compares assistant professors and full professors in neurosurgical specialties. It was observed that assistant professors had the highest concentration of spine and endovascular subspecialties when compared to full professors [2]. This suggests that spine surgery is emerging, and younger surgeons are choosing this path. Rising demand due to an aging population and an increase in degenerative spine disease is also contributing to the increase in spine subspecializations. By 2030, one in six people globally will be over 60 years old. In the United States, it is expected that those aged 65 and above will comprise 22% of the population [7]. Chronic back pain alone affects 45.6% of adults 65 and older [8]. In the US, the clinical and economic burden of spine care is rapidly expanding and warrants the need for fellowship-trained neurosurgeons. 

However, the location of neurosurgical spine fellowships limits the geographic availability of these specialists, despite the widespread need for them due to both the prevalence of spine disease and the aging population. According to the North American Spine Society Fellowship Directory, there are only three spine fellowship programs in the state of Florida. These programs are the West Florida Spine Fellowship, the University of Miami/Jackson Memorial Medical Center Spine Fellowship, and the Florida Orthopaedic Institute Spine Fellowship, each offering two positions per year [9]. There is an emerging need for additional fellowships as subspecializing has become more popular throughout surgery in general. As neurosurgeons pursuing spine fellowships become more common, factors influencing fellowship are considered. This includes academic versus private career goals, geographic location, and job demand. 

Despite this evidence, there is a lack of state-level and regional analysis. Specifically, there is a lack of data regarding the Florida workforce and spine-focused neurosurgeons. There is also a lack of studies on female neurosurgeons or those who hold osteopathic doctoral degrees (DO) compared to those who hold a doctor of medicine (MD). Our study aims to identify changes in the number of Florida-based neurosurgeons pursuing spine-focused fellowship training in the past 60 years. We aim to identify the percentage of neurosurgeons pursuing spine fellowships who are DOs or females by conducting a retrospective cross-sectional study on neurosurgeons specializing in spine, comparing those with fellowship training and those without. Outcomes analyzed include fellowship training status, current academic practice, geographic distribution, degree type, and professional affiliation between fellowship-trained and non-fellowship-trained neurosurgeons.

## Materials and methods

This retrospective cross-sectional study collected demographic data and bibliometric information on neurosurgeons specializing in spine surgery, comparing those with fellowship training and those without. Data were collected between February 2026 and April 2026 and represented a snapshot of actively practicing neurosurgeons during this time period. 

Inclusion and exclusion criteria

Physician data were gathered from the American Association of Neurological Surgeons (AANS) and the Florida Department of Health databases [10,11]. A comprehensive list of neurosurgeons was collected, and surgeons were included if they were board-certified or board-eligible neurosurgeons who identified spine surgery as a primary area of practice, had an active license, and were currently practicing in Florida. Neurosurgeons without sufficient publicly available data regarding training were excluded from the analysis (Table 1).

**Table 1:** Inclusion and exclusion criteria for spine neurosurgeons.

Inclusion criteria (total n = 113)	Exclusion criteria
Board-certified neurosurgeons	Not enough publicly available data (n = 61)
Spine surgery identified as primary area of practice	Null and void licenses (n = 96)
Active medical license	Delinquent or inactive licenses (n = 69)
Currently practicing in FL	Retired or deceased (n = 25)

The following data were collected for the spine neurosurgeons: name, sex, graduation year from residency and fellowship if applicable, MD or DO status, fellowship program attended, current practice status (academic vs. non-academic), and AANS affiliation. Biological sex was determined based on publicly available institutional profiles and professional listings. Academic status was defined as a faculty appointment at an accredited medical school or an affiliated teaching hospital. Program data were also collected for further analysis. This included geographic and demographic information, such as city and state for residency and fellowship programs.

The primary outcome of interest was the change in the proportion of fellowship-trained spine neurosurgeons over time. Secondary outcomes included differences in geographic distribution, degree type, sex, and professional affiliation between fellowship-trained and non-fellowship-trained neurosurgeons. The academic practice setting was examined as an additional descriptive outcome. The data were analyzed using descriptive statistics and linear regression analysis using the Excel Analysis ToolPak to identify trends. A Bonferroni correction was applied to account for multiple comparisons across the regression analyses. The figures and tables were created using Google Sheets and Excel. 

Missing data were handled by excluding variables with incomplete entries on a per-analysis basis. To ensure accuracy, data extraction was performed systematically by two reviewers and cross-checked for consistency by the third reviewer. This study utilized publicly available data and did not involve human subjects or identifiable private information; it was exempt from institutional review board approval.

## Results

A total of 113 spine-specialized neurosurgeons with active medical licenses and currently practicing were identified in the state of Florida at the time of this study in April 2026. Out of a total of 113, only two (1.77%) spine neurosurgeons are female, and four (3.54%) have a DO degree. The proportion of DO-trained spine neurosurgeons showed a non-significant upward trend over time (slope = 0.75% per five-year period, 95% CI: -0.02% to 1.52%, R² = 0.320, p = 0.055). Furthermore, female representation among spine neurosurgeons showed no statistically significant change over time (slope = -0.12% per five-year period, 95% CI: -1.14% to 0.90%, R² = 0.007, p = 0.800). 

**Figure 1:**
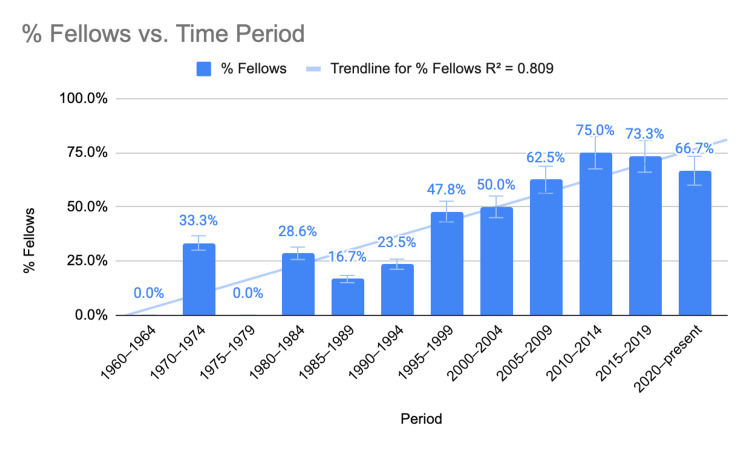
Percentage of fellowship-trained spine neurosurgeons in Florida.

Among spine neurosurgeons in Florida, the proportion with spine fellowship training varied over time. In 1960-1964 and 1975-1979, none (0.00%; n = 0) had completed a spine fellowship, while 33.3% (n = 1) had done so in 1970-1974. The proportion was 28.57% (n = 2) in 1980-1984 and 16.67% (n = 2) in 1985-1989. An overall increase was observed in subsequent years: 23.53% (n = 4) in 1990-1994, 47.83% (n = 11) in 1995-1999, 50.00% (n = 4) in 2000-2004, 62.50% (n = 5) in 2005-2009, and 75.00% (n = 6) in 2010-2014. The highest percentage was observed in 2015-2019 at 73.33% (n = 11), followed by 66.67% (n = 6) in 2020-present (Figure 1).

There has been a statistically significant increase in the rate of obtaining spine fellowship over the time periods (slope = 6.69% per five-year period, 95% CI: 4.4-9.0%, R² = 0.808, p < 0.001). In addition, 67.26% (n = 76) of neurosurgeons are working in academic institutions, while the rest 32.74% (n = 37) are in non-academic settings. Among fellowship-trained surgeons with available data (n = 47), 57.45% (n = 27) practiced in academic settings compared to 70.91% (n = 39) of non-fellowship-trained surgeons (n = 55); this difference was not statistically significant (χ² = 1.465, p = 0.226). Moreover, 71.68% (n = 81) of neurosurgeons had an AANS affiliation, while the rest (28.32%; n = 32) were not a part of the AANS. It has been calculated that AANS affiliation rates increased significantly over time (slope = 5.43% per five-year period, 95% CI: 1.98-8.88%, R² = 0.552, p = 0.006). After Bonferroni corrections (adjusted α = 0.0125), both the fellowship rate trend (p < 0.001) and AANS affiliation trend (p = 0.024) remained statistically significant, while DO proportion (p = 0.055) and female representation (p = 0.800) remained non-significant. A summary table was created for spine neurosurgeons’ characteristics mentioned below (Table 2). 

**Table 2:** Cohort characteristics of Florida spine neurosurgeons by residency graduation period.

Graduation period (residency)	Surgeons (n)	Fellowship trained (n)	Non-fellowship (n)	% fellowship trained	Female (n)	% Female	DO (n)	% DO	AANS affiliated (n)	% AANS affiliated
1960-1964	1	0	1	0.00%	0	0.00%	0	0.00%	0	0.00%
1970-1974	3	1	2	33.30%	0	0.00%	0	0.00%	1	33.30%
1975-1979	2	0	2	0.00%	0	0.00%	0	0.00%	1	50.00%
1980-1984	7	2	5	28.60%	1	14.30%	0	0.00%	6	85.70%
1985-1989	12	2	10	16.70%	0	0.00%	0	0.00%	8	66.70%
1990-1994	17	4	13	23.50%	0	0.00%	1	5.90%	12	70.60%
1995-1999	23	11	12	47.80%	0	0.00%	0	0.00%	16	69.60%
2000-2004	8	4	4	50.00%	1	12.50%	1	12.50%	4	50.00%
2005-2009	8	5	3	62.50%	0	0.00%	0	0.00%	5	62.50%
2010-2014	8	6	2	75.00%	0	0.00%	0	0.00%	7	87.50%
2015-2019	15	11	4	73.30%	0	0.00%	1	6.70%	13	86.70%
2020-pres.	9	6	3	66.70%	0	0.00%	1	11.10%	8	88.90%
Total	113	52	61	46.00%	2	1.80%	4	3.50%	81	71.70%

For the further fellowship analysis, 23.53% (n = 12) of Florida-based spine neurosurgeons obtained their fellowship training in Florida, most commonly at the University of Miami/Jackson Health System. The rest of the fellowships were distributed as follows 72.55% (n = 37) have completed fellowships in other states of the US, and the 3.92% (n = 2) outside of the US (Table 3). 

**Table 3:** Demographics of fellowship locations for Florida spine neurosurgeons.

Fellowship location	Florida	Rest of the US	Abroad
n	12	37	2
%	23.53%	72.55%	3.92%

The residency programs completed by neurosurgeons were analyzed based on location. 27.68% (n = 31) of spine-neurosurgeons have completed their residency in Florida, while 66.07% (n = 74) completed residency in other states in the US, and 6.25% (n = 7) have completed it outside of the US (Table 4).

**Table 4:** Demographics of residency locations for Florida spine neurosurgeons.

Residency location	Florida	Rest of the US	Abroad
n	31	74	7
%	27.68%	66.07%	6.25%

## Discussion

Over the span of the past 60+ years, Florida has seen an increase in the number of neurosurgeons choosing to pursue a spine fellowship. The greatest escalation in this trend was seen from 2015 to 2019. This surge likely stems from the growing complexities of spinal procedures and increasing indications for spinal surgery within medicine. With a growing focus on spinal procedures in the United States, spinal fellowships are becoming more popular and competitive [2]. Academic appointments have also expanded over time, independent of fellowship status. Although the number of neurosurgeons pursuing fellowship stranded from a 73.30% to a 66.70% between 2015-2019 and the current decade, this difference could likely be attributed to the small cohort of neurosurgeons present and is not in itself a large enough group to judge for statistical significance. 

Geographical analysis of the training sites within Florida revealed that the majority of neurosurgeons who chose to specialize in spinal procedures did so outside of the state, with the only in-state institutions being West Florida, Florida Orthopedic Institute, and the University of Miami Health System. With the centralization of fellowships in specific areas of the state and country, it becomes crucial to acknowledge the impact on the localization of the workforce. This can impact the long-term distribution of neurosurgeons and academic institutions, preventing underserved areas from receiving the same level of care and specialty focus. A previous research study did a geospatial analysis on neurological care and access, and found there to be a significant difference in the ease of access to care between urban centers and rural areas [12]. Furthermore, another nationwide geospatial analysis evaluating robotic spinal procedure access found that a patient seeking care only had to travel 57 miles if near an urban area, as compared to 222 miles if in a rural area [13]. With this difficulty in accessing neurological and spinal care, patients in underserved areas are likely to face worse health outcomes, with distance being a strong indicator of safe surgical outcomes. 

In addition, despite the growth in pursuance of spine fellowships, Florida still seems to face a gender and degree disparity in terms of who is choosing to pursue a spine fellowship. In Florida, there are only two females specializing in spine, in comparison to the 111 neurosurgeons who are male. Furthermore, there is also a gap in the total number of female physicians who choose to pursue a career in neurosurgery. As of 2019, a study found that in the United States, women made up approximately 8.2% of practicing neurosurgeons [14]. With this wide variance in gender, it becomes important to understand the reasons why this gap exists. Some of the main barriers women face in pursuing neurosurgery include lack of mentorship, hesitancy due to lifestyle implications, and discouragement from entering the field [2,15,16]. One of the lifestyle reasons that women cite for not pursuing neurosurgery is the inflexibility of their schedule regarding pregnancy, giving birth, and childcare [17]. By focusing on evolving the roles to be more inclusive of women, the male-heavy focus can be neutralized and become less intimidating for women choosing to pursue a career in neurosurgery and spine. 

Along with a disparity in gender within the field, there is also a discrepancy regarding the type of medical degree. Of the 113 practicing spine neurosurgeons in Florida, only four hold a doctorate in osteopathic medicine. Despite osteopathic medicine having a strong focus on skeletal anatomy, specifically the spine, there is an extremely low number of DO physicians who pursue neurosurgery, particularly in the field of spine care. This wide gap indicates an underlying problem within the field of neurosurgical medicine. For osteopathic physicians, there are a greater number of barriers that prevent them from pursuing a career in neurosurgery. There is not only a reduced focus on neurosurgical training and research, but there is also a limited number of affiliated residency pipelines and greater competition within the field. Furthermore, the successful match rate from 2020 to 2023 found that for MD students, as compared to DO students, is significantly lower, at 74.82% versus 30.88%, which can intimidate the students possibly interested in applying [18]. Research also plays a pivotal role in neurosurgery residency applications; however, NIH only provides 0.1% of research funding towards Osteopathic schools, preventing students from being able to expand their research [19,20]. With these factors in play, it becomes difficult for an osteopathic student to gain the skills and knowledge to successfully apply and match into neurosurgery. 

Limitations

This study’s ability to determine causality is limited by its retrospective cross-sectional design. In addition, it relies on publicly available information from databases, such as the AANS and the Florida DOH. These databases may report incomplete, outdated, or inconsistent information, especially about fellowship training, work settings, and academic roles. Limited or incomplete online profiles could lead to misclassification or omission of some physicians, introducing selection bias. Excluding individuals with insufficient information may also reduce the generalizability of the results. Furthermore, some early five-year cohorts contained very small sample sizes, which could reflect a disproportionate leverage on the regression line, therefore limiting the reliability of the trend analyses from those periods.

Due to these methodological constraints, the results should be interpreted as descriptive and associative rather than causal. Biological sex was inferred from names, pronouns, and photos from publicly available profiles rather than self-report, which may not accurately reflect self-identified gender. Unmeasured confounders, such as individual career preferences, institutional opportunities, research productivity, and mentorship, were not captured and may affect both the likelihood of pursuing fellowship training and an academic career. Larger prospective or longitudinal studies with more comprehensive datasets are needed to better understand the relationship between fellowship training and career outcomes in spine neurosurgery.

## Conclusions

This study shows a significant rise in the proportion of fellowship-trained spine neurosurgeons in Florida over the past several decades. Our findings parallel national trends and indicate an expanding trend of subspecialization within neurosurgical training. Despite this progression, women and DO neurosurgeons face a disconnect from national trends. To close this gap and achieve greater uniformity, it is important to identify the barriers preventing them from becoming spine neurosurgeons. Targeted efforts to expand equitable training opportunities, strengthen exposure and mentorship earlier in medical training, and address system-wide barriers that limit diversity within neurosurgery are needed. Promoting inclusion and improving access to training and care will be essential to attaining a more representative workforce and maximizing patient outcomes throughout diverse populations.

Our study further demonstrates that nearly one in four of the neurosurgeons are trained in a spine fellowship within the state. Among fellowship-trained surgeons with available data, 57.45% (n = 27) practiced in academic settings; however, this proportion did not differ significantly from non-fellowship-trained surgeons (70.91%, n = 39; p = 0.226) and should be interpreted as a descriptive observation rather than a causal association. This trend also introduces possible challenges. Future studies researching the accessibility of neurosurgical care within the state and the nation are needed to better understand the impact on patients in rural communities.
